# Supplementary material for: A “Green” Homogenate Extraction Coupled with UHPLC-MS for the Rapid Determination of Diterpenoids in Croton Crassifolius
Source: Molecules. 2019 Feb 15;24(4):694. doi: 10.3390/molecules24040694 (PMC6413027; doi:10.3390/molecules24040694)
Supplement: Supplementary file 1 [file molecules-24-00694-s001.zip › molecules-443449-SI.pdf]

Article

A "Green" Homogenate Extraction Coupled with UHPLC-MS for the Rapid Determination of Diterpenoids in *Croton Crassifolius*

Jin-Long Tian, Chi Shu, Ye Zhang, Hui-Jun Cui, Xu Xie, Xu-Long Ran, Tian-Shun Chen, Zhi-Huan Zang, Jian-Guo Liu and Bin Li *

College of Food Science, Shenyang Agricultural University, Shenyang 110866, China; sweet_vs_sweet@163.com (J.-L.T.); 2018500024@syau.edu.cn (C.S.); zysyau@163.com (Y.Z.); chj0423@163.com (H.-J.C.); syauxiexu@163.com (X.X.); ranxulong12@163.com (X.-L.R.); cts166282@163.com (T.-S.C.); m139983676611@163.com (Z.-H.Z.); 18547552365@163.com (J.-G.L.)

* Correspondence: libinsyau@163.com

Figure S1 The isolation procedure for clerodane diterpenoids **1-9** from *C. crassifolius*.

Figure S2 ^{1}H NMR spectrum (400 MHz, $CDCl_3$) of compound **1**

Figure S3 ^{13}C NMR spectrum (100 MHz, $CDCl_3$) of compound **1**

Figure S4 ^{1}H NMR spectrum (400 MHz, $CDCl_3$) of compound **2**

Figure S5 ^{13}C NMR spectrum (100 MHz, $CDCl_3$) of compound **2**

Figure S6 ^{1}H NMR spectrum (400 MHz, $CDCl_3$) of compound **3**

Figure S7 ^{13}C NMR spectrum (100 MHz, $CDCl_3$) of compound **3**

Figure S8 ^{1}H NMR spectrum (400 MHz, $CDCl_3$) of compound **4**

Figure S9 ^{13}C NMR spectrum (100 MHz, $CDCl_3$) of compound **4**

Figure S10 ^{1}H NMR spectrum (400 MHz, $CDCl_3$) of compound **5**

Figure S11 ^{13}C NMR spectrum (100 MHz, $CDCl_3$) of compound **5**

Figure S12 ^{1}H NMR spectrum (400 MHz, $CDCl_3$) of compound **6**

Figure S13 ^{13}C NMR spectrum (100 MHz, $CDCl_3$) of compound **6**

Figure S14 ^{1}H NMR spectrum (400 MHz, $CDCl_3$) of compound **7**

Figure S15 ^{13}C NMR spectrum (100 MHz, $CDCl_3$) of compound **7**

Figure S16 ^{1}H NMR spectrum (400 MHz, $CDCl_3$) of compound **8**

Figure S17 ^{13}C NMR spectrum (100 MHz, $CDCl_3$) of compound **8**

Figure S18 ^{1}H NMR spectrum (400 MHz, $CDCl_3$) of compound **9**

Figure S19 ^{13}C NMR spectrum (100 MHz, $CDCl_3$) of compound **9**

Figure S20 The effect of diterpenoids **1–9** on VEGF release.

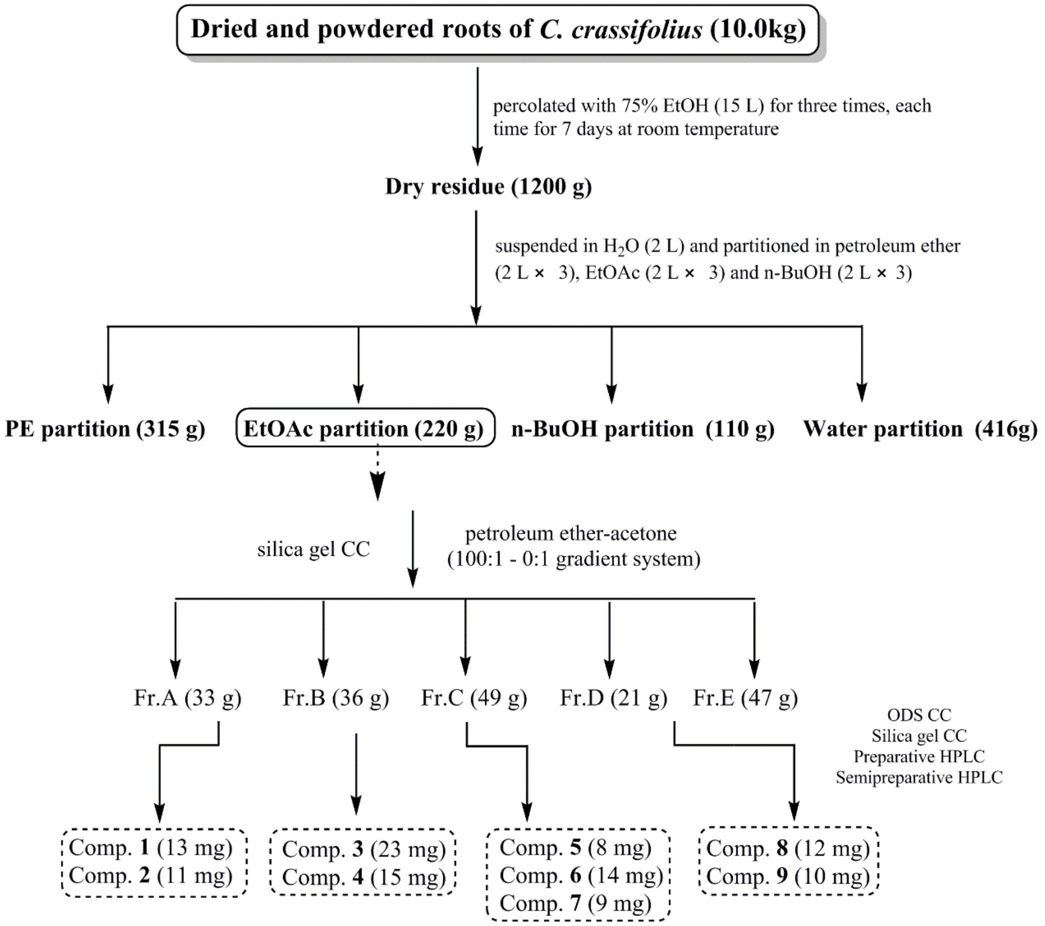

Figure S1 The isolation procedure for clerodane diterpenoids **1-9** from *C. crassifolius*.

The dried and powdered roots of *C. crassifolius* (10.0 kg) were percolated with 75% EtOH (15 L) for three times, each time for 7 days at room temperature. The ethanol extract was concentrated under reduced pressure to yield a brownish-dark crude extract (1200 g), The extracts were combined and concentrated under reduced pressure to leave a residue, which was then suspended in H_2O (2 L) and partitioned in petroleum ether (2 L × 3), EtOAc (2 L × 3) and *n*-BuOH (2 L × 3). The EtOAc partition (220 g) was applied to silica gel CC, eluting with petroleum ether-acetone (100:1–0:1 gradient system). Five crude fractions (A-E) were obtained by TLC analysis. Fr. A (33 g) was further chromatographed on silica gel column with petroleum ether-acetone (50:1 to 0:1) to provide two fractions (Frs. A1 (6.3 g); A2 (4.1 g); A3 (4.5 g); A4 (5.3 g); A5 (3.7 g); A6 (4.9 g)), among which Fr. A2 (4.1 g) was subjected to an ODS gel column (MeOH/H_2O; 60:40; 80:20; 100:0, v/v) to provide three fractions to yield three fractions: Frs. A2-1 (240 mg); A2-2 (1.1 g), A2-3 (1.7 g); then Frs. A4-1 (240 mg) was subjected to semipreparative HPLC eluted with aqueous methanol (75%, v/v; 2.5 ml/min) to yield compounds **1** (13 mg; t_R 17.7 min), **2** (11 mg; t_R 21.8 min). Fr. B (36 g) was subjected to an ODS gel column (MeOH/H_2O; 60:40; 80:20; 100:0, v/v) to provide three fractions to yield three fractions: Frs. B1 (5.6 g); B2 (126 mg), B3 (1.7 g), then Frs. B2 (126 mg) was subjected to semipreparative HPLC eluted with aqueous methanol (79%, v/v; 2.5 ml/min) to yield compounds **3** (23 mg; t_R 12.1 min), **4** (15 mg; t_R 24.6 min). Fr. C (49 g) was further chromatographed on CC (petroleum ether-acetone; 20:1; 10:1; 5:1; 2:1; 1:1, v/v) to provide five

fractions: Frs. C1 (77 mg); C2 (45 mg); C3 (118 mg); C4 (1.76 g); C5 (1.4 g), and then C3 (118 mg) was subjected to semipreparative HPLC eluted with aqueous methanol (70%, v/v; 2.5 ml/min) to yield compounds **5** (8 mg; t_R 14.7 min), **6** (14 mg; t_R 22.6. min), **7** (9 mg; t_R 28.1 min). Then Fr. D (21 g) was further chromatographed on CC (petroleum ether-acetone; 20:1; 10:1; 5:1; 2:1; 1:1, v/v) to provide four fractions: Frs. D1 (452 mg); D2 (168 mg); D3 (5.8 mg); D4 (2.1 g), D2 (168 mg) was further chromatographed on Sephadex LH-20 (MeOH/H_2O; 80:20, v/v) to provide three fractions: Frs. D2-1 (27 mg); D2-2 (24 mg); D2-3 (49 mg) and D2-3 (49 mg) was subjected to semipreparative HPLC eluted with aqueous methanol (78%, v/v; 2.5 ml/min) to yield compounds **8** (12 mg; t_R 15.7 min), and compounds **9** (10 mg; t_R 25.1 min).

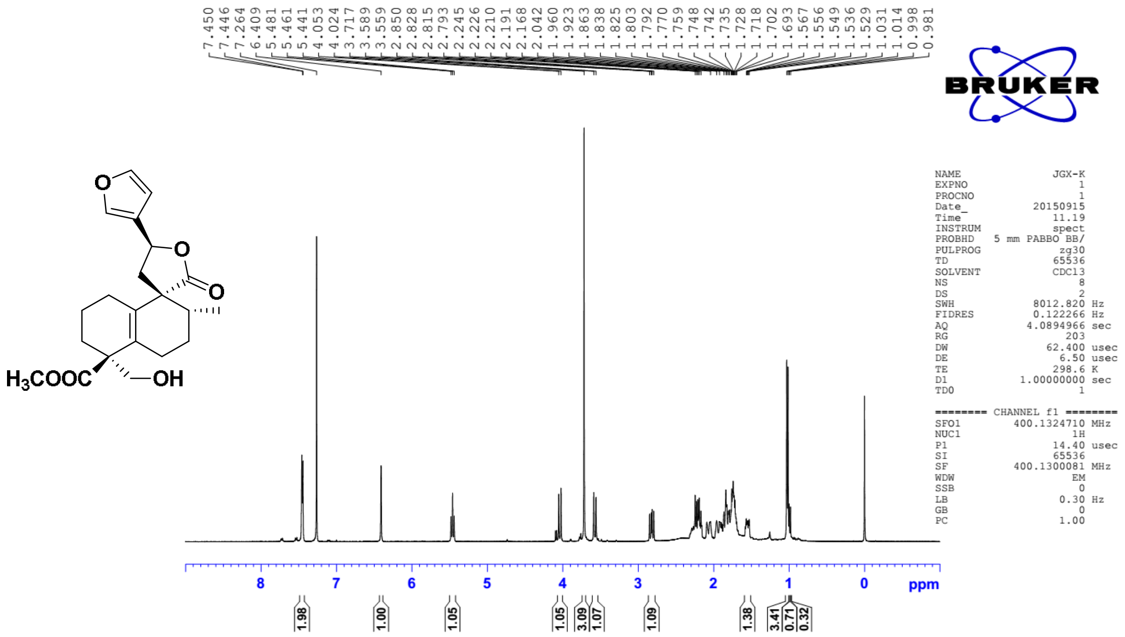

Figure S2. ^{1}H NMR spectrum (400 MHz, $CDCl_3$) of compound **1**

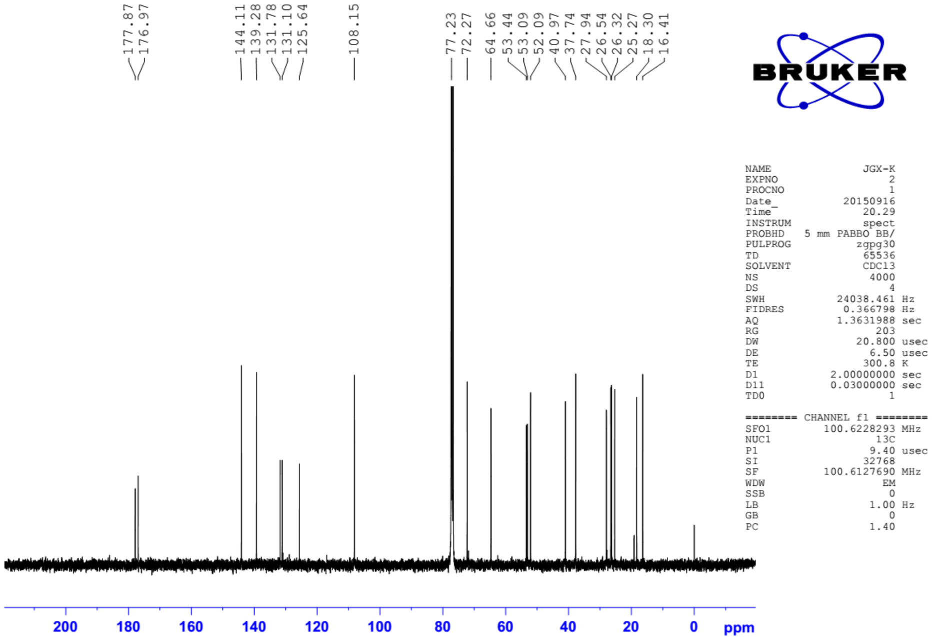

Figure S3. ^{13}C NMR spectrum (100 MHz, $CDCl_3$) of compound **1**

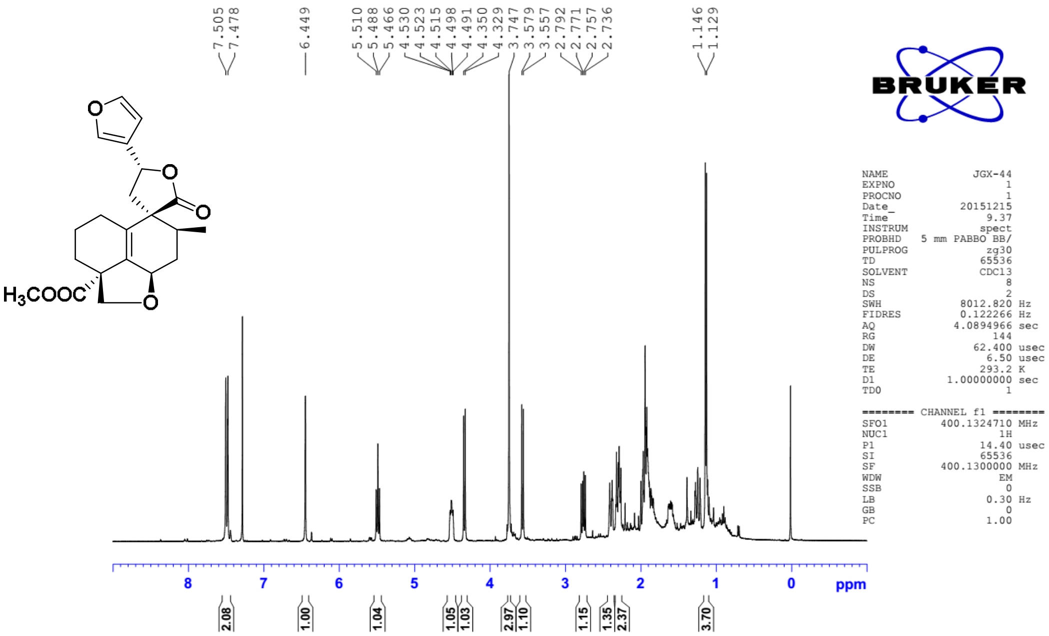

Figure S4. ^{1}H NMR spectrum (400 MHz, $CDCl_3$) of compound **2**

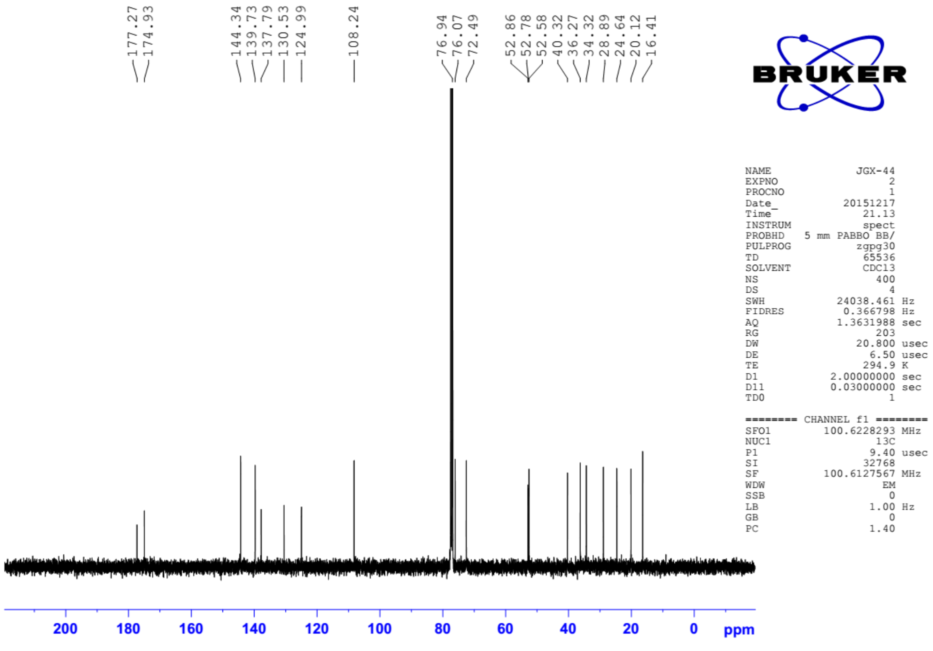

Figure S5. ^{13}C NMR spectrum (100 MHz, $CDCl_3$) of compound **2**

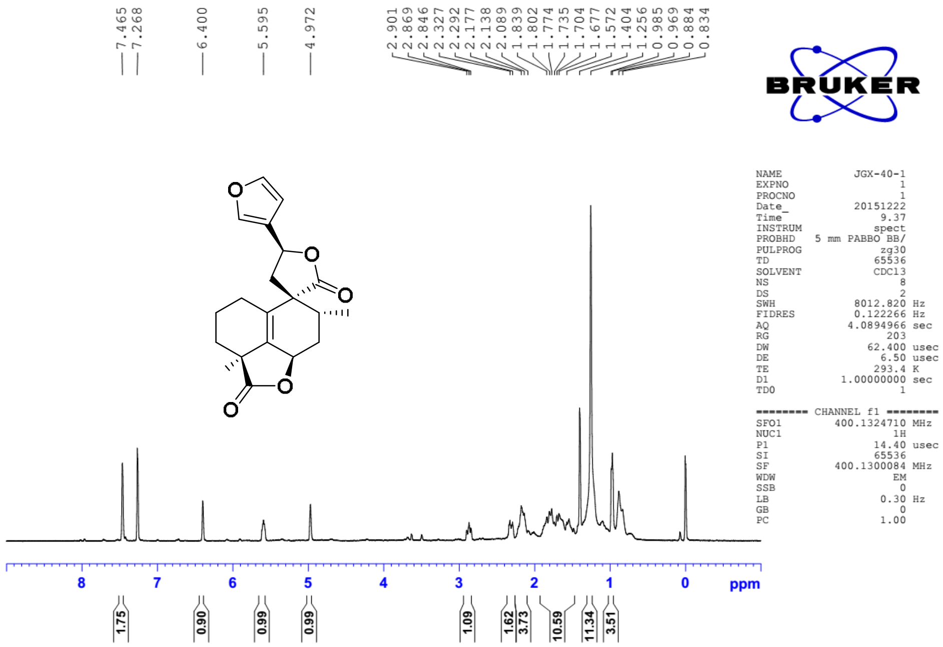

Figure S6. ^{1}H NMR spectrum (400 MHz, $CDCl_3$) of compound **3**

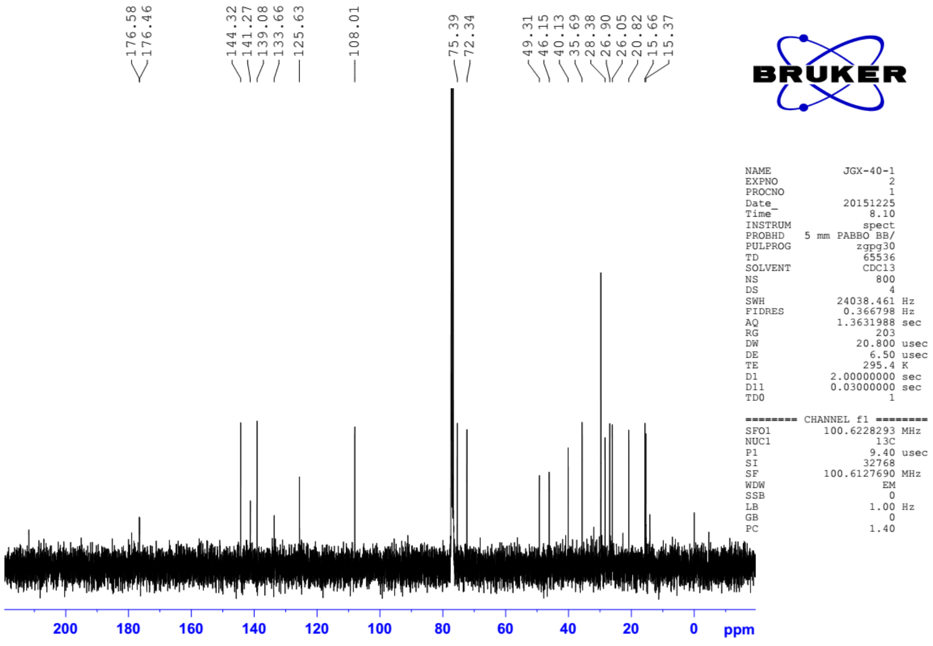

Figure S7. ^{13}C NMR spectrum (100 MHz, $CDCl_3$) of compound **3**

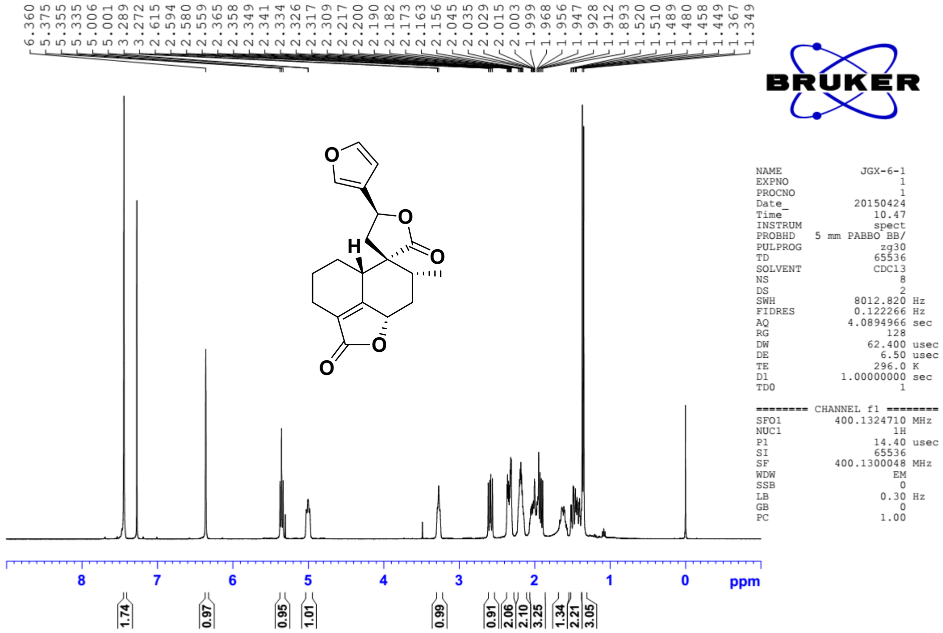

Figure S8. ^{1}H NMR spectrum (400 MHz, $CDCl_3$) of compound **4**

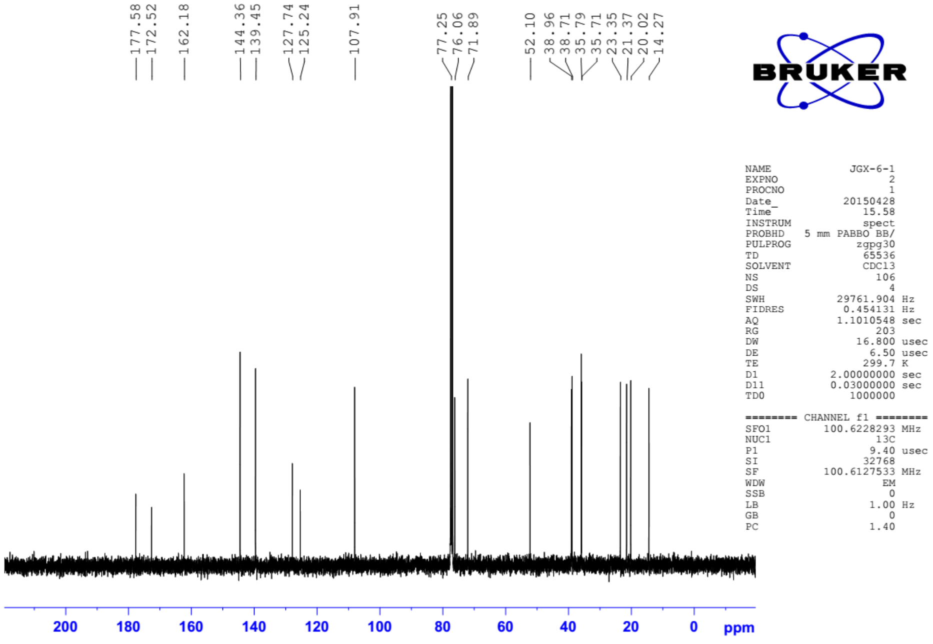

Figure S9 ^{13}C NMR spectrum (100 MHz, $CDCl_3$) of compound **4**

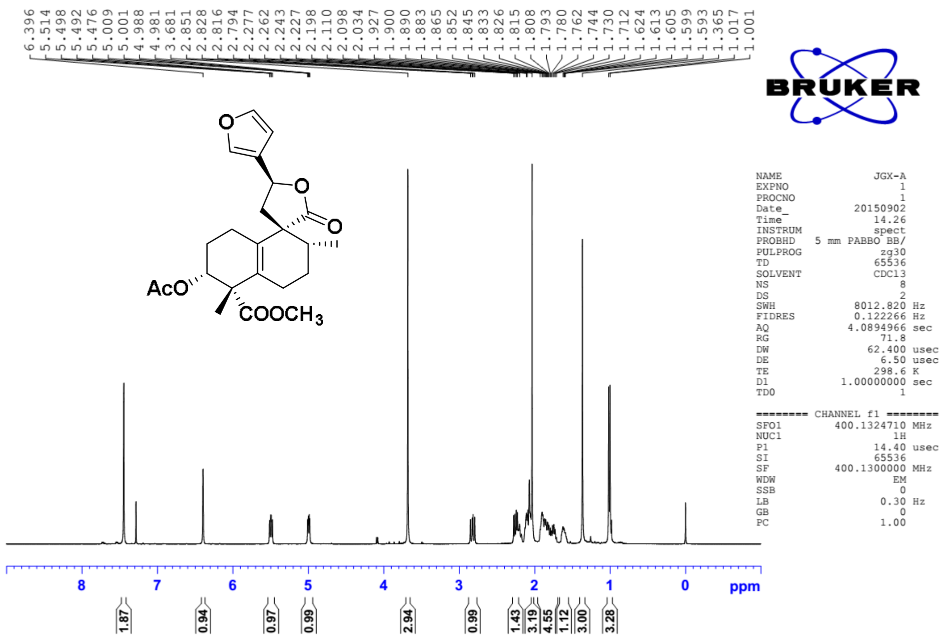

Figure S10. ^{1}H NMR spectrum (400 MHz, $CDCl_3$) of compound **5**

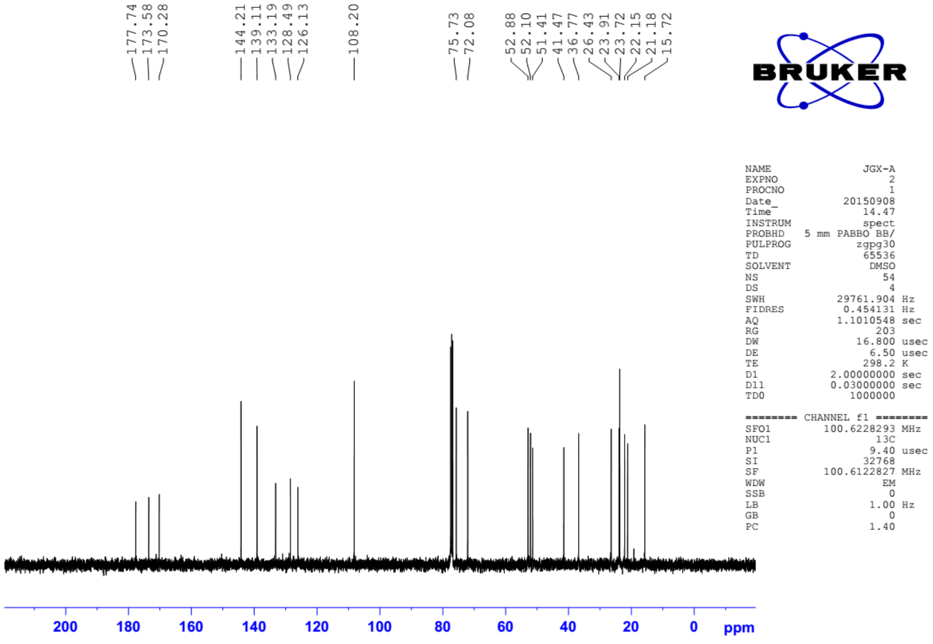

Figure S11 ^{13}C NMR spectrum (100 MHz, $CDCl_3$) of compound **5**

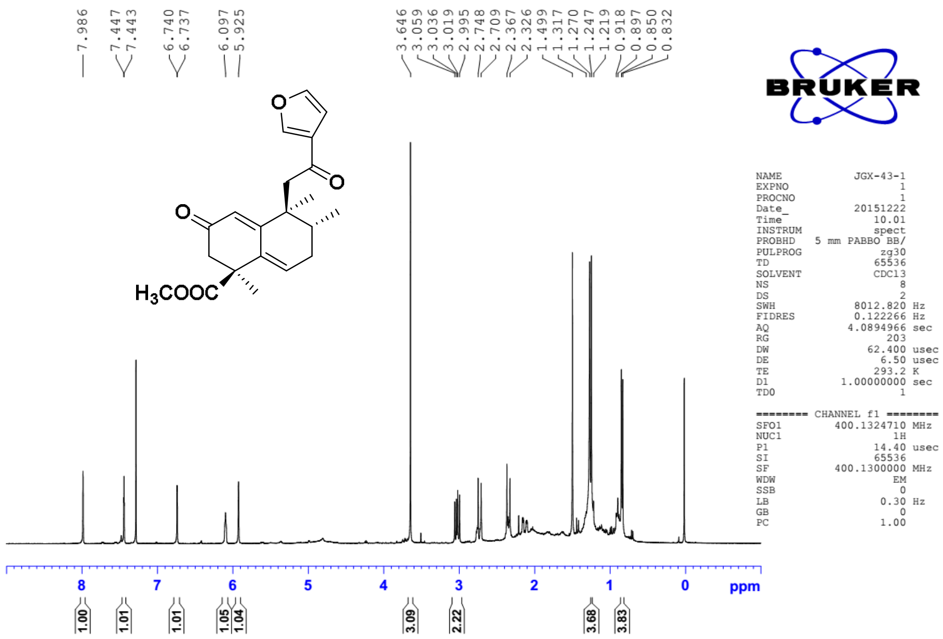

Figure S12. ^{1}H NMR spectrum (400 MHz, $CDCl_3$) of compound **6**

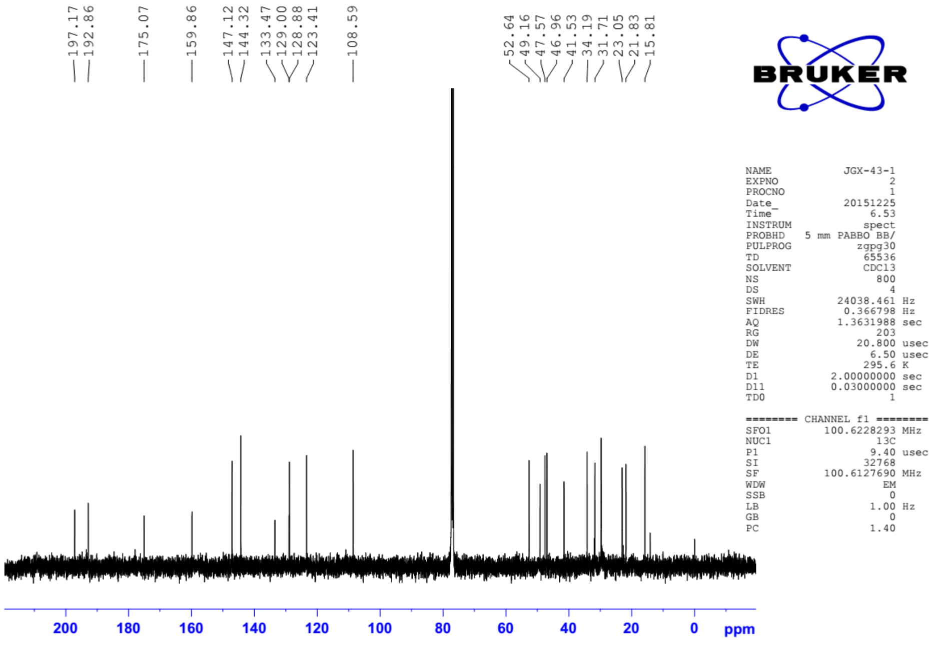

Figure S13 ^{13}C NMR spectrum (100 MHz, $CDCl_3$) of compound **6**

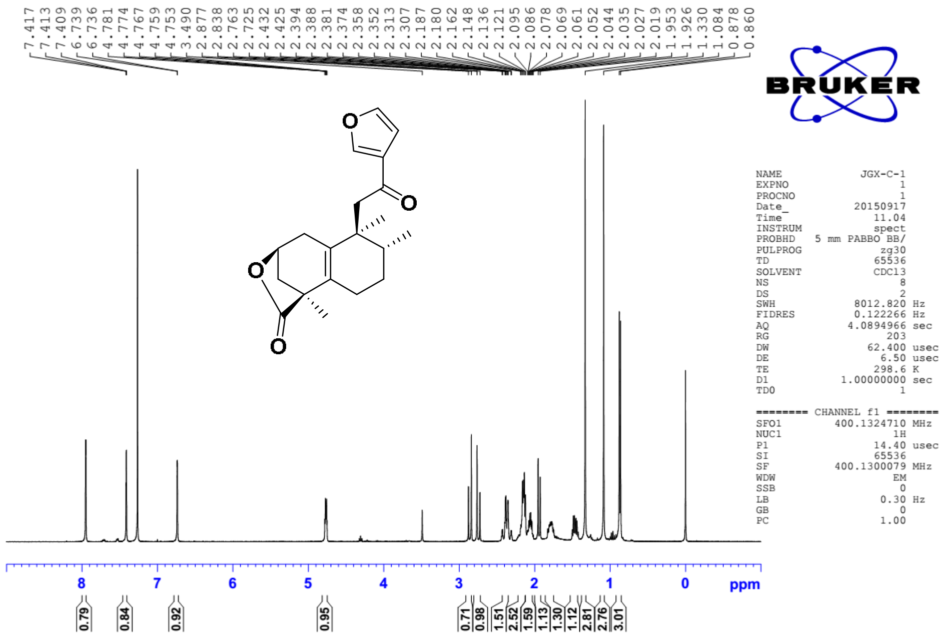

Figure S14. ^{1}H NMR spectrum (400 MHz, $CDCl_3$) of compound **7**

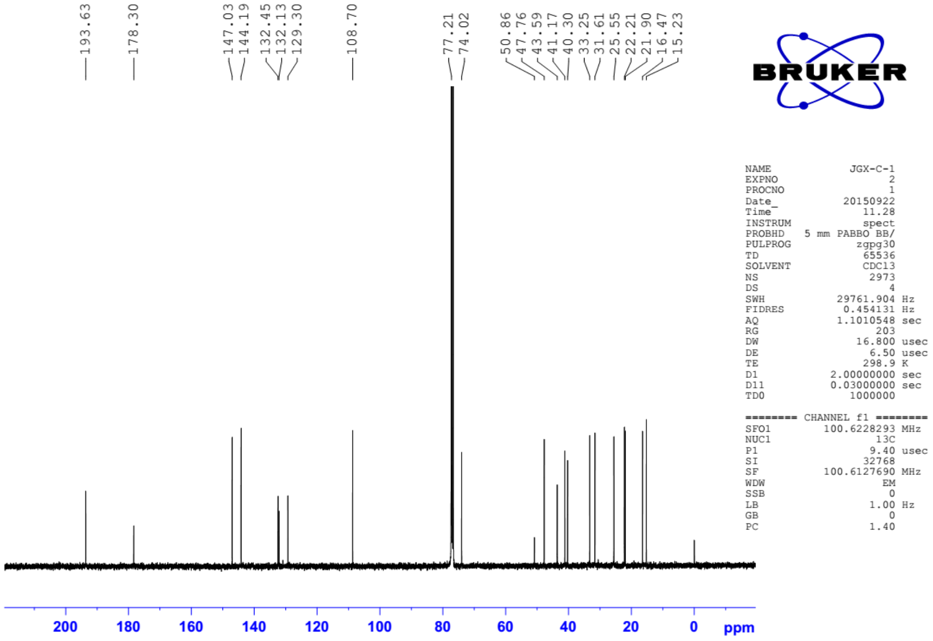

Figure S15 ^{13}C NMR spectrum (100 MHz, $CDCl_3$) of compound **7**

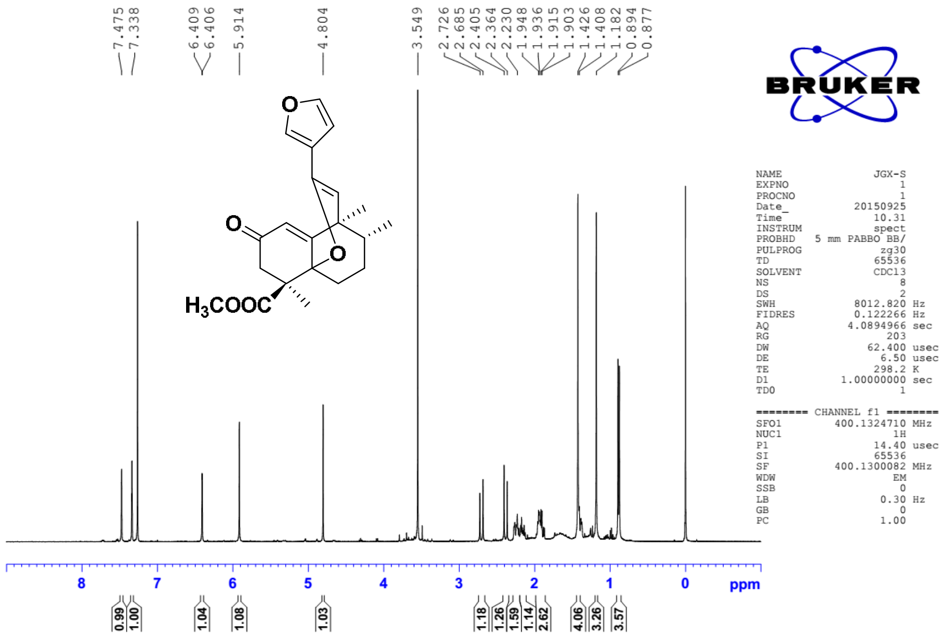

Figure S16. ^{1}H NMR spectrum (400 MHz, $CDCl_3$) of compound **8**

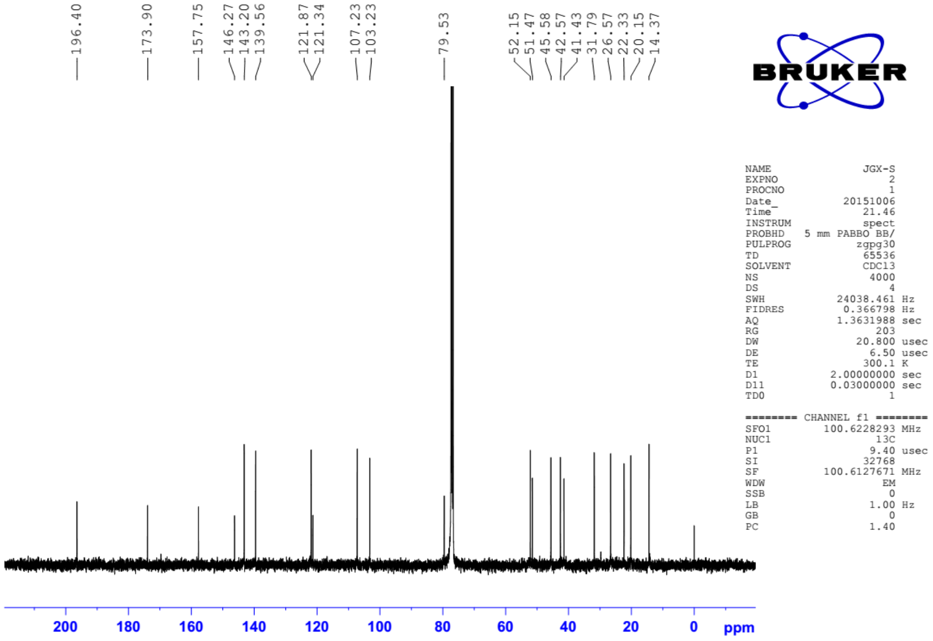

Figure S17^{13}C NMR spectrum (100 MHz, $CDCl_3$) of compound **8**

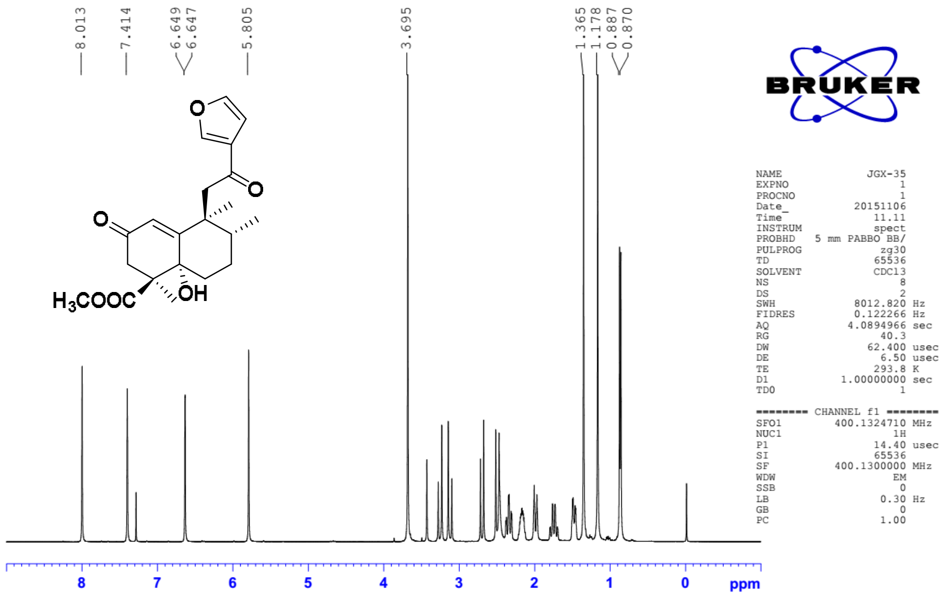

Figure S18. ^{1}H NMR spectrum (400 MHz, $CDCl_3$) of compound **9**

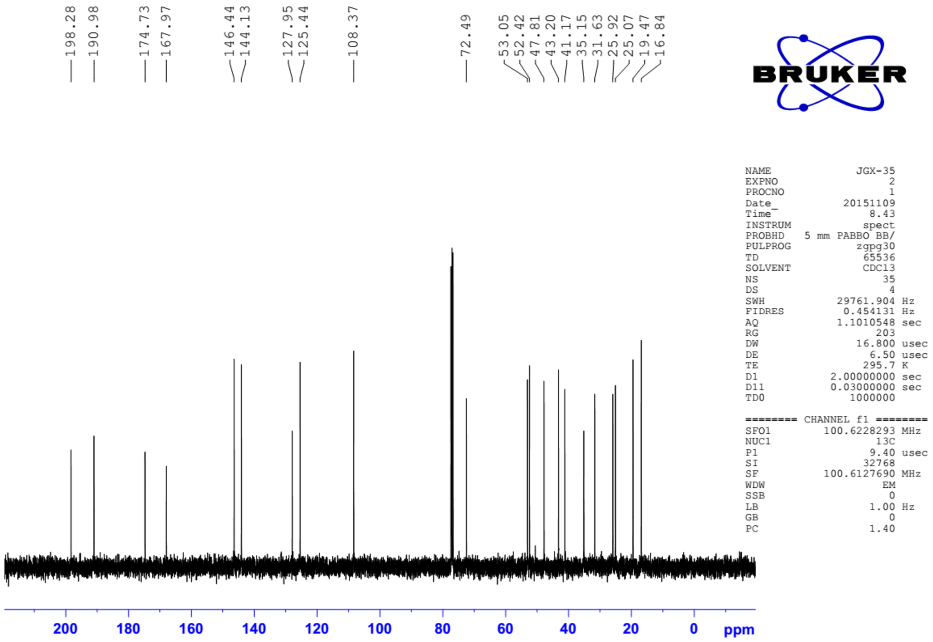

Figure S19 ^{13}C NMR spectrum (100 MHz, $CDCl_3$) of compound **9**

Compound 1 (Crassifolin L) [1]:
^{1}H-NMR (400MHz, $CDCl_3$): *δ* 2.03 (2H, m, H-1), 1.84 (1H, m, H-2), 1.70 (1H, m, H-2), 2.17 (1H, m, H-3), 1.84 (1H, m, H-3), 2.20 (1H, m, H-6), 1.52 (1H, m, H-6), 1.84 (2H, m, H-7), 1.70 (1H, m, H-8), 2.79 (1H, dd, *J* = 8.4 Hz, 13.6 Hz, H-11), 2.18 (1H, m, H-11), 5.43 (1H, dd, *J* = 8.4, 8.3 Hz), 6.37 (1H, s, H-14), 7.41 (1H, s, H-15), 7.42 (1H, s, H-16), 0.98 (3H, d, *J* = 6.8 Hz), 4.00 (1H, d, *J* = 12.0 Hz), 3.54 (1H, d, *J* = 12.0 Hz), 3.68 (3H, s, H-21).
^{13}C-NMR (100MHz, $CDCl_3$): *δ* 25.4 (C-1), 18.4 (C-2), 28.1 (C-3), 53.2 (C-4), 131.2 (C-5), 26.7 (C-6), 26.5 (C-7), 37.9 (C-8), 53.6 (C-9), 131.9 (C-10), 41.1 (C-11), 72.5 (C-12), 125.8 (C-13), 108.3 (C-14), 144.3 (C-15), 139.4 (C-16), 16.6 (C-17), 177.1 (C-18), 64.8 (C-19), 178.2 (C-20), 53.6 (C-21).
Compound 2 (Crassifolin D) [2]:
^{1}H-NMR (400MHz, $CDCl_3$): *δ* 2.44 (1H, m, H-1), 1.82 (1H, m, H-1), 1.91 (1H, m, H-2), 1.61 (1H, m, H-2), 2.35 (1H, m, H-3), 1.21 (1H, m, H-3), 4.48 (2H, m, H-6), *δ*1.88 (2H, m, H-7), 1.87 (1H, m, H-8), 2.78 (1H, dd, *J* = 14.1, 8.2 Hz, H-11), 2.33 (1H, m, H-11), 5.66 (1H, dd, *J* = 8.7, 8.5 Hz, H-12), 6.53 (1H, m, H-14), 7.56 (1H, m, H-15), 7.65 (1H, m, H-16), 1.11 (1H, d, *J* = 6.1 Hz, H-17), 4.28 (1H, d, *J* = 8.7 Hz, H-19), 3.53 (1H, d, *J* = 8.8 Hz, H-19), 3.72 (1H, s, H-OMe).
^{13}C-NMR (100MHz, $CDCl_3$): *δ* 25.5 (C-1), 21.2 (C-2), 30.1 (C-3), 54.1 (C-4), 138.3 (C-5), 77.4 (C-6), 35.5 (C-7), 37.2 (C-8), 54.4 (C-9), 132.7 (C-10), 40.9 (C-11), 74.5 (C-12), 126.4 (C-13), 109.5 (C-14), 145.7 (C-15), 141.8 (C-16), 16.9 (C-17), 176.5 (C-18), 77.9 (C-19), 179.7 (C-20), 53.1 (C- OMe).
Compound 3 (neoclerodan-5,10-en-19,6b;20,12-diolide) [3]:
^{1}H-NMR (400MHz, $CDCl_3$): *δ* 0.89 (3H, d, *J* = 6:6 Hz, CH3-17), 1.31 (3H, s, CH_3-18), 2.33 (1H, dd, *J* = 14, 5.6 Hz, H-11), 2.79 (1H, dd, *J* = 14, 9 Hz, H-11), 4.9 (1H, dd, *J* = 3:7, 1.4 Hz, H-6), 5.5 (1H, dd, *J* = 9, 5.6 Hz, H-12), 6.32 (1H, br. s., H-14), 7.38 (2H, m, H-15 and H-16).
^{13}C-NMR (100MHz, $CDCl_3$): *δ* 26.8 (C-1), 20.7 (C-2), 28.3 (C-3), 46.1 (C-4), 141.1 (C-5), 75.3 (C-6), 25.9 (C-7), 35.6 (C-8), 49.3 (C-9), 133.5 (C-10), 40.0 (C-11), 72.3 (C-12), 125.5 (C-13), 107.9 (C-14), 139.0 (C-15), 144.2 (C-16), 15.3 (C-17),15.5 (C-18), 176.4 (C-19), 176.5 (C-20).
Compound 4 (Teucvin) [4]:
^{1}H-NMR (400MHz, $CDCl_3$): *δ* 2.20 (1H, m, H-1), 1.45 (1H, m, H-1), 1.98 (1H, m, H-2), 1.54 (1H, m, H-2), 2.20 (1H, m, H-3), 2.15 (1H, m, H-3), 4.77 (1H, dd, *J* = 9.8, 7.8 Hz, H-6), 2.20 (1H, m, H-6), 1.86 (1H, m, H-8), 2.66 (1H, m, H-10), 2.53 (1H, d, *J* = 8.3Hz, H-11), 5.43 (1H, dd, *J* = 8.3, 8.4 Hz, H-6), 6.37 (1H, s, H-14), 7.44 (1H, s, H-15), 7.45 (1H, s, H-16), 1.05 (1H, d, *J* = 6.7 Hz, H-17).
^{13}C-NMR (100MHz, $CDCl_3$): 24.8 (C-1), 21.7 (C-2), 19.6 (C-3), 126.6 (C-4), 161.3 (C-5), 78.2 (C-6), 35.3 (C-7), 36.0 (C-8), 53.5 (C-9), 42.1 (C-10), 40.9 (C-11), 71.8 (C-12), 125.0 (C-13), 107.9 (C-14), 144.3 (C-15), 139.5 (C-16), 17.0 (C-17), 172.9 (C-18), 175.5 (C-19).
Compound 5 (Crassifolin F) [1]:
^{1}H-NMR (400MHz, $CDCl_3$): *δ* 1.88 (2H, m, H-1), 2.04 (2H, m, H-2), 4.95 (1H, m, H-3), 2.34 (2H, m, H-6), 1.72 (1H, m, H-7), 1.65 (1H, m, H-7), 1.84 (1H, m, H-8), 2.86 (1H, dd, *J* = 14.0, 8.9, H-11), 2.32 (1H, m, H-11), 5.61 (1H, dd, *J* = 8.6, 6.4 Hz, H-7), 7.55 (1H, s, H-15), 7.59 (1H, s, H-16), 0.99 (1H, d, *J* = 6.8 Hz, H-17), 1.36 (3H, s, H-19), 2.01 (3H, s, H-22), 3.66 (3H, s, H-OMe).
^{13}C-NMR (100MHz, $CDCl_3$): *δ* 25.1 (C-1), 24.8 (C-2), 77.2 (C-3), 52.8 (C-4), 134.5 (C-5), 24.6 (C-6), 27.6 (C-7), 38.2 (C-8), 54.6 (C-9), 130.2 (C-10), 42.2 (C-11), 74.2 (C-12), 127.6 (C-13), 109.5 (C-14), 145.7 (C-15), 141.0 (C-16), 16.3 (C-17), 172.2 (C-18), 22.7 (C-19), 180.5 (C-20), 175.4 (C-21), 21.2 (C-22), 52.4 (C-OMe).
Compound 6 (Crassifolin C) [1]:
^{1}H-NMR (400MHz, $CDCl_3$): *δ* 5.83 (1H, s, H-1), 2.25 (1H, m, H-3), 2.92 (1H, m, H-3), 6.01 (1H, s, H-6), 2.04 (1H, m, H-7), 2.65 (1H, m, H-7), 2.26 (1H, m, H-8), 2.66 (1H, m, H-11), 2.94 (1H, m, H-11), 6.66 (1H, m, H-14), 7.35 (1H, s, H-15), 7.92 (1H, s, H-16), 0.75 (3H, d, *J* = 6.8 Hz, H-7), 1.41 (3H, s, H-7), 1.15 (3H, s, H-7), 3.54 (3H, s, H-OMe).
^{13}C-NMR (100MHz, $CDCl_3$): *δ* 123.7 (C-1), 197.2 (C-2), 47.7 (C-3), 49.3 (C-4), 133.5 (C-5), 128.9 (C-6), 31.7 (C-7), 34.4 (C-8), 41.6 (C-9), 159.9 (C-10), 47.0 (C-11), 192.9 (C-12), 129.1 (C-13), 109.2 (C-14), 145.1 (C-15), 147.3 (C-16), 16.0 (C-17), 175.1 (C-18), 23.1 (C-19), 21.4 (C-20), 52.7 (C-OMe).
Compound 7 (6-[2-(furan-3-yl)-2-oxoethyl]-1,5,6-trimethyl-10-oxatricyclo[7.2.1.02,7]dodec-2(7)-en-11-one) [5]:
^{1}H-NMR (400MHz, $CDCl_3$): *δ* 2.33 (1H, dd, *J* = 17.9, 2.7 Hz, H-1), 2.40 (1H, m, H-1), 4.76 (1H, m, H-2), 1.93 (1H, m, H-3), 2.13 (1H, m, H-3), 2.14 (2H, m, H-6), 1.46 (1H, m, H-7), 1.79 (1H, m, H-7), 2.04 (1H, m, H-8), 2.74 (1H, d, *J* = 15.5 Hz, H-11), 2.85 (1H, d, *J* = 15.5 Hz, H-11), 6.73 (1H, s, H-14), 7.41 (1H, s, H-15),7.95 (1H, s, H-16), 0.86 (3H, d, *J* = 7.0, H-17), 1.32 (3H, s, H-19), 1.07 (3H, s, H-20).
^{13}C-NMR (100MHz, $CDCl_3$): *δ* 31.6 (C-1), 74.0 (C-2), 41.1 (C-3), 43.6 (C-4), 132.1 (C-5), 22.2 (C-6), 25.5 (C-7), 33.2 (C-8), 40.3 (C-9), 132.4 (C-10), 47.7 (C-11), 193.6 (C-12), 129.3 (C-13), 108.7 (C-14), 144.2 (C-15), 147.6 (C-16), 15.2 (C-17), 178.3 (C-18), 16.5 (C-19), 21.9 (C-20).

Compound 8 (9-(furan-3-yl)-2,7,13-trimethyl-4-oxo-10-oxatricyclo[5.3.3.01,6]trideca-5,8-diene-2-carboxylate) [5]:
^{1}H-NMR (400MHz, $CDCl_3$): δ 5.90 (1H, s, H-1), 2.38 (1H, d, *J* = 16.3 Hz, H-3), 2.39 (1H, d, *J* = 16.3 Hz, H-3), 1.89 (1H, dd, *J* = 13.3, 4.8 Hz, H-6), 2.34 (1H, dd, *J* = 13.3, 4.8 Hz, H-6), 1.40 (1H, m, H-7), 2.16 (1H, m, H-7), 1.95 (1H, m, H-8), 4.80 (1H, m, H-11), 6.40 (1H, s, H-14), 7.33 (1H, s, H-15), 7.47 (1H, s, H-16), 0.88 (3H, d, *J* = 7.0 Hz, H-17), 1.42 (3H, s, H-19), 1.17 (3H, s, H-19), 3.54 (3H, s, H-OMe).
^{13}C-NMR (100MHz, $CDCl_3$): δ 121.8 (C-1), 196.4 (C-2), 45.5 (C-3), 51.4 (C-4), 79.5 (C-5), 31.7 (C-6), 26.5 (C-7), 42.5 (C-8), 41.4 (C-9), 157.7 (C-10), 103.2 (C-11), 146.2 (C-12), 121.3 (C-13), 107.2 (C-14), 143.2 (C-15), 139.2 (C-16), 14.3 (C-17), 173.9 (C-18), 20.1 (C-19), 22.3 (C-20), 52.1 (C-OMe).
Compound 9 (Chettaphanin-I) [6]:
^{1}H-NMR (400MHz, $CDCl_3$): δ 5.81 (1H, s, H-1), 2.51 (1H, m, H-2), 2.71 (1H, m, H-1), 2.37 (1H, m, H-6), 1.99 (1H, m, H-6), 1.75 (1H, m, H-7), 1.50 (1H, m, H-7), 2.40 (1H, m, H-8), 3.12 (1H, d, *J* = 19 Hz, H-11), 3.28 (1H, d, *J* = 19 Hz, H-11), 6.64 (1H, s, H-14), 7.41 (1H, s, H-15), 8.00 (1H, s, H-16), 0.88 (1H, d, *J* = 7.1 Hz, H-17), 1.39 (3H, s, H-18), 1.18 (3H, s, H-20), 3.70 (3H, s, H-OMe).
^{13}C-NMR (100MHz, $CDCl_3$): δ 125.7 (C-1), 190.1 (C-2), 53.1 (C-3), 43.4 (C-4), 72.8 (C-5), 32.1 (C-6), 25.3 (C-7), 35.4 (C-8), 41.7 (C-9), 167.2 (C-10), 48.0 (C-11), 197.4 (C-12), 128.2 (C-13), 108.4 (C-14), 144.0 (C-15), 149.6 (C-16), 16.7 (C-17), 25.8 (C-18), 174.5 (C-19), 19.5 (C-20), 52.27 (C-OMe).

Reference

[1] Wang J. J., Chung H. Y., Zhang Y. B., Li G. Q., Li Y. L., Huang W. H., Wang G. C. (2016). Diterpenoids from the roots of Croton crassifolius and their anti-angiogenic activity. *Phytochemistry*, 122, 270-275.

[2] Wang G. C., Li J. G., Li G. Q., Xu J. J., Wu X., Ye W. C., Li Y. L. (2012). Clerodane Diterpenoids from *Croton crassifolius*. J. Nat. Prod., 75, 2188-2192.

[3] Kapingu M. C., Guillaume D., Mbwambo Z. H., Moshi M. J., Uliso F. C., Mahunnah R. L. A. (2000). Diterpenoids from the roots of *Croton macrostachys*. Phytochemistry, 54, 767-770.

[4] Mbwambo Z. H., Foubert K., Chacha M., Kapingu M. C., Magadula J. J., Moshi M. M., Lemière F., Goubitz K., Fraanje J., Peschar R., Vlietinck A., Sandra A., Pieters L. (2009). New Furanoditerpenoids from Croton jatrophoides. Planta Med., 75, 262–267.

[5] Kanlayavattanakul M., Ruangrungsi N., Watanabe T., Kawahata M., Therrien B., Yamaguchi K., Ishikawa, T. (2005). ent-Halimane Diterpenes and a Guaiane Sesquiterpene from *Cladogynos orientalis*. J. Nat. Prod., 68, 7-10.

[6] Boonyarathanakornkit L., Che C. T., Fong H. H. S., Farnsworth N. R. (1988). Constituents of *Croton crassifolius* Roots. Planta Med., 1, 61-63.

The effect of diterpenoids **1**–**9** on VEGF release

Angiogenesis can influence tumor development. VEGF is a biomarker of tumor metastasis and angiogenesis. It is the most special and strong strongest angiogenic growth factor, the over expression of which occurs in cancer cell lines, like HepG2. In this study, the effect of diterpenoids **1**–**9** on the release of VEGF in HepG2 was tested through an ELISA method. The results showed that after 72 h cultured, compounds **1**–**9** at the dose of 50 μM suppressed the release of VEGF in HepG2 cells (**Figure S20**).

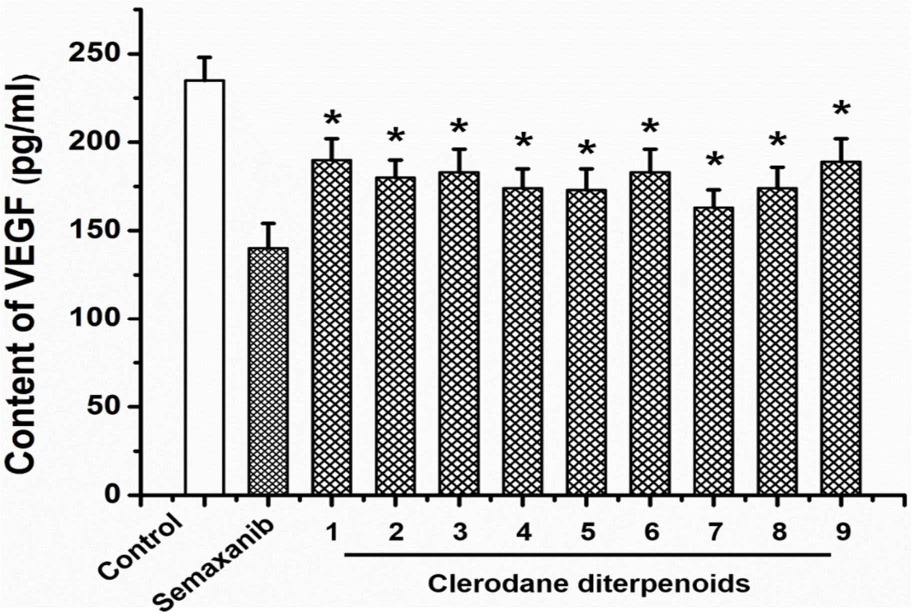

Figure S20. Diterpenoids **1**-**9** significantly suppressed the release of VEGF in HepG2 cells. *p <0.05 vs. control group.
